# Insights Into the Structural and Functional Evolution of Plant Genomes Afforded by the Nucleotide Sequences of Chromosomes 2 and 4 of *Arabidopsis Thaliana*

**DOI:** 10.1002/(SICI)1097-0061(200004)17:1<1::AID-YEA3>3.0.CO;2-V

**Published:** 2000

**Authors:** Ian Bancroft

**Affiliations:** John Innes Centre Norwich Research Park Colney Norwich NR4 7UH UK

## Abstract

The rapidly accumulating genome sequence data from the plant *Arabidopsis thaliana* allows more detailed analysis of genome content and organisation than ever bafore possible in plants. The genome shows a surprisingly high level of genetic redundancy, with as many as 75% of gene products showing signficant homology to another protien of *A. thaliana.* Many duplicated genes occur in arrays of conserved order and indicate that *A. thaliana* is likely to have had a tetraploid ancestor. Analysis of the divergence of duplicated genome segments leads to the prediction of two major modes of plant genome evolution: macro-scale duplication and rearrangement of chromosomes and micro-scale translocation, duplication and loss of individual genes or small groups of genes.


**Yeast**
*Yeast* 2000; **17**: 1–5.

**Review Article**

### Insights into the structural and functional evolution of plant genomes afforded by the nucleotide sequences of chromosomes 2 and 4 of *Arabidopsis thaliana*

Ian Bancroft*
*John Innes Centre, Norwich Research Park, Colney, Norwich NR4 7UH, UK*

**Correspondence to:*
*I. Bancroft, John Innes Centre, Norwich Research Park, Colney, Norwich NR4 7UH, UK.*
*E-mail: ian.bancroft@bbsrc.ac.uk*

Received: 24 January 2000
Accepted: 1 February 2000

### Abstract

**The rapidly accumulating genome sequence data from the plant *Arabidopsis thaliana* allows more detailed analysis of genome content and organisation than ever before possible in plants. The genome shows a surprisingly high level of genetic redundancy, with as many as 75% of gene products showing significant homology to another protein of *A. thaliana*. Many duplicated genes occur in arrays of conserved order and indicate that *A. thaliana* is likely to have had a tetraploid ancestor. Analysis of the divergence of duplicated genome segments leads to the prediction of two major modes of plant genome evolution: macro-scale duplication and rearrangement of chromosomes and micro-scale translocations, duplication and loss of individual genes or small groups of genes. Copyright © 2000 John Wiley & Sons, Ltd.**

**Keywords:** ***Arabidopsis thaliana*; *Brassica*; plant genome evolution; genome duplication; polyploidy; genome rearrangements**

The small plant *Arabidopsis thaliana*, which is a member of the Brassicaceae, has been widely adopted as a model for the study of many aspects of plant biology. It shows all of the developmental and physiological characteristics typical of flowering plants, and exhibits straightforward diploid genetics. Because of its small genome size, approximately 130 Mbp, it was chosen as the subject of the first plant genome sequencing project. It is widely anticipated that the complete nucleotide sequence of the genome of *A. thaliana* will greatly enhance our understanding of plant genome organization and will allow the identification of all of the genes of a typical flowering plant. These data will be a vital component underpinning new and efficient strategies for the improvement of crops.

It is expected that the complete sequence of the genome of *A. thaliana* will be available by the end of 2000. The sequences of chromosomes 2 and 4, complete and contiguous except for gaps corresponding to the centromere-containing regions, were reported recently [8,10]. The combined total of 37 Mbp of sequence data contain 30–35% of all of the protein-coding genes of *A. thaliana*. In all, 7781 protein-coding genes were identified, an average of one every 4.76 kb. The mean statistics for gene structure are: 4.91 exons of 276 bp and 3.91 introns of 183 bp, resulting in a span per gene of 2071 bp, or 44% of the genome. The remainder constitutes regulatory, non-translated and intergenic sequences. Thus, the genome of *A. thaliana* has evolved to be very gene-rich and shows similar mean density and exon number per gene as that of the 97 Mb genome of the nematode worm *Caenorhabditis elegans* [3]. However, the larger genome of *A. thaliana* should compose 22 000–26 000 genes, significantly more than that of *C. elegans,* which is predicted to contain 19 000. Although *C. elegans* appears a more complex organism, the greater number of genes in *A. thaliana* underlines the great complexity of the environmental sensing and responses required by a sessile organism, and the number of genes needed to facilitate the wide range of secondary metabolism conducted by plants. For both chromosomes 2 and 4, the densities of genes identified and matched to expressed sequence tags

Copyright © 2000 John Wiley & Sons, Ltd.

(ESTs) is uniform along the majority of the chromosome arms. Close to the centromeres, however, the gene density drops as the proportions of transposable elements and other repetitive sequences increase greatly relative to their very low levels along the chromosome arms.

The common occurrence of tandemly repeated genes was noted during pilot-scale sequencing [1]. Analysis of the larger data set suggests that 12–15% of all genes of *A. thaliana* may be the result of tandem duplications. More surprising was the observed extent of the occurrence of homologous genes at distant chromosomal locations and the identification of numerous segmental duplications. Extrapolation to account for the presently incomplete status of the *A. thaliana* genome sequence suggests that as many as 75% of gene products might show significant homology to another protein of *A. thaliana*. The majority of the corresponding homologous genes are scattered apparently randomly across the genome, but many genes were identified as being in conserved arrays, indicating segmental duplications. Further studies have indicated that as much as 60% of the genome is arranged in large blocks that appear to be the result of ancient duplications (M. Delseney, G. Blanc, A. Barakat, R. Guyot and R. Cooke, personal communication). Some are very large, e.g. a 4.6 Mbp region of chromosome 2 is duplicated on chromosome 4, with 430 of 1100 genes (39%) being conserved [8]. Other, smaller segments can show higher degrees of conservation. For example, Figure 1 illustrates a 47-gene segment of chromosome 4, for which 33 genes (69%) are conserved, in perfect colinear order, on chromosome 5.

The genes identified in the nucleotide sequences of chromosomes 2 and 4 were analysed for putative function, on the basis of homology matches of their protein products with those of genes of known function. Overall, 55% of genes were found to have sufficient homology to genes of known function to allow categorization and putative broad function to be determined. Many genes, particularly those involved in transcription, protein synthesis, cell growth and division, intracellular transport and signal transduction defined a substantial eukaryote-specific set of genes. We have no idea of the functions of the 45% of genes not categorized. More than 65% of the predicted proteins on chromosome 2 do not show significant similarity to those specified by any other completed genome, suggesting they are unique to plants [8].

What do the sequences of chromosomes 2 and 4 of *A. thaliana* tell us about plant genome evolution? Many comparative genetic mapping studies have revealed conservation of genome organization between related species, e.g. between tomato and potato [15] and between grasses [11]. There is, however, evidence of extensive genome rearrangements between other related species. The 'diploid' *Brassica* species, including *Brassica nigra*, are degenerate hexaploids, with three copies of a unit genome resembling that of *A. thaliana* [5,6,2]. On the basis of comparative genetic mapping, there were estimated to have been ca. 90 rearrangements between the genomes *of A. thaliana* and *B. nigra* [6], which diverged 10–35 million years ago [12,6]. This rate of rearrangement within the Brassicaceae contrasts with that observed in the grasses, where only 19 linkage blocks in an ancestral cereal genome can be reassembled to represent the genomes of six present-day species [11], some of which are polyploids, and which diverged as much as 60 million years ago [18,9]. The majority of flowering plants are polyploid, and polyploidy is thought to play a major role in the evolution of higher plants [7]. The extensive duplications identified in the *A. thaliana* genome suggest that *A. thaliana* had a tetraploid ancestor. The observation that the duplicated segments in *A. thaliana* are extensively broken-up and dispersed across the genome is in accordance with the hypothesis that rearrangement of chromosomes may have an important role in the stabilization of polyploid genomes in plants [7]. The duplication represented in Figure 1, which is one of the best conserved, is of relatively ancient origin. The same duplication is present in all unit copies of the genome of *Brassica oleracea*, thus dating its occurrence to at least 10–35 million years ago, although several individual genes are absent from corresponding positions (C. O'Neill and I. Bancroft, unpublished).

The *A. thaliana* sequence data indicate that a variety of micro-scale evolutionary mechanisms have operated in addition to macro-scale duplication and rearrangement. Analysis of the chromosome 4 genes not conserved in the chromosome 5 segment, as illustrated in Figure 1, indicated that three genes (at4g17260, at4g17540 and at4g17590) have homologues scattered around the genome, suggesting that translocation of individual genes

Copyright © 2000 John Wiley & Sons, Ltd. 

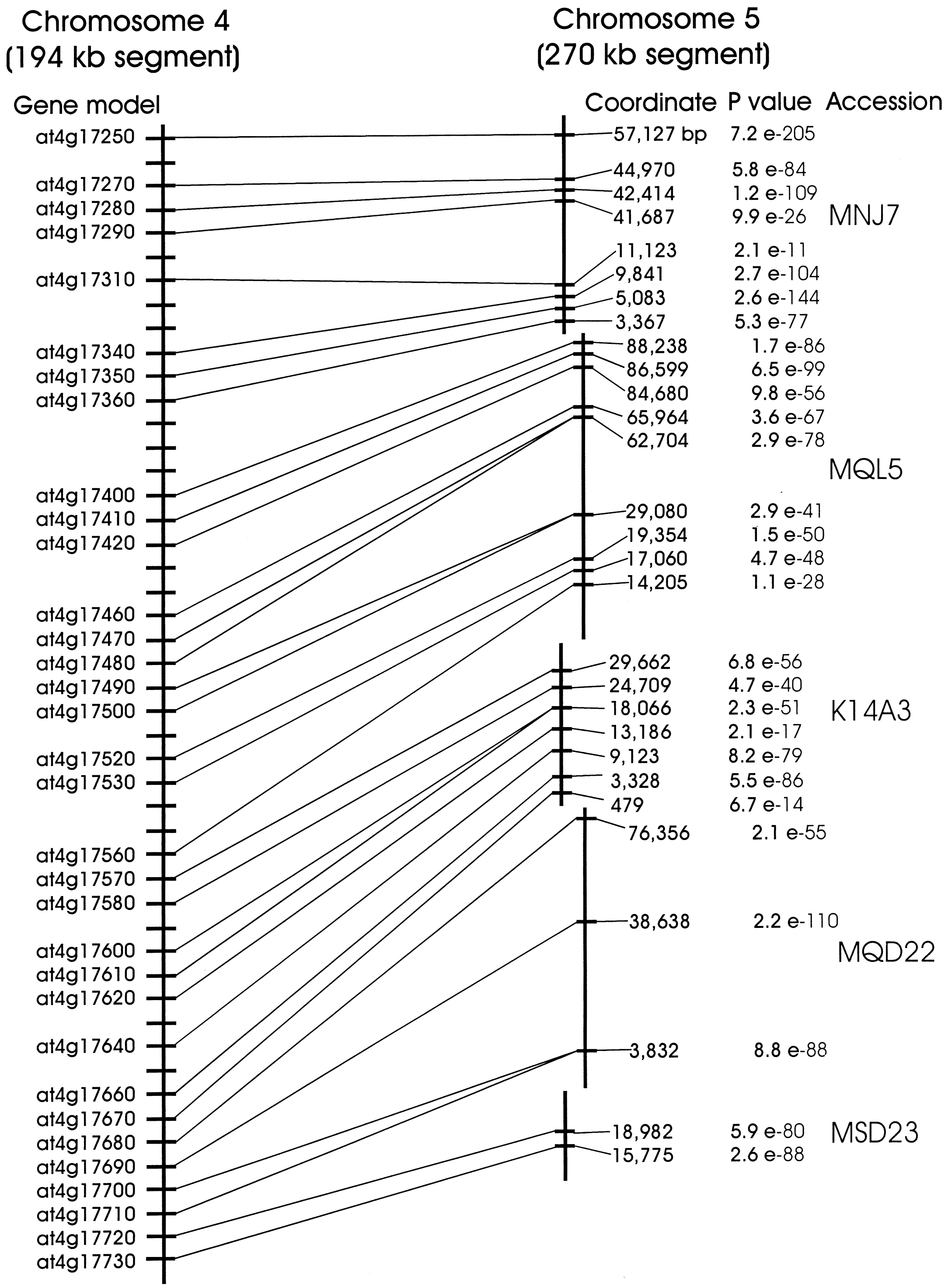

**Figure 1.** Segmental duplications on chromosomes 4 and 5 of *A. thaliana*. Nucleotide sequences representing all modelled genes on chromosome 4 between at4g15970 and at4g17810 were obtained from the MIPS website (**http://www.mips.biochem.mpg.de/**) and used to conduct BLASTN analysis on the MIPS server to a database of *A. thaliana* genomic DNA sequences. For the region containing at4g17250 to at4g17730, all gene models with a homologue in a contiguous segment of chromosome 5 sequence data, represented in accessions MNJ7, MQL5, K14A3, MQD22 and MSD23, are listed. Also shown are the end coordinates and P values for the most highly homologous segment encompassed by each gene model

Copyright © 2000 John Wiley & Sons, Ltd.

may have occurred. Ten genes have no homologues elsewhere in the genome, indicating deletion or extensive divergence of gene sequences. A cluster of three genes, at4g17380, at4g17381 and at4g17340 (the last of which also has a conserved homologue on chromosome 5), have homologues (P values from BLASTN analysis of 2.4 e-24, 1.5 e-28 and 2.6e-23, respectively) ca. 250 kb away on chromosome 4. This suggests that short-range, but not tandem, small-scale duplication events occur. Detailed physical mapping and sequencing has been conducted for putatively homoeologous segments of the genomes of *A. thaliana* (the chromosome 4 region illustrated in Figure 1) and rice, which diverged ca. 200 million years ago [18]. These studies provided no evidence for a corresponding duplication in the rice genome, but indicated that many micro-scale changes to gene content and position have occurred during the divergence of these species, resulting in extensive alteration of fine-scale genome organization. The result is that only a framework consisting of a few, largely non-rearranged, conserved genes is discernible today (van Dodeweerd *et al.* [16]; G. Murphy, C. Hall and I. Bancroft, unpublished).

Taken together, the data comparing gene organization both within the *A. thaliana* genome and between that of *A. tha*liana, *B. oleracea* and rice, suggest that the construction of unified genetic maps will be more complex than had been speculated [13]. It appears that macroscopic rearrangement of chromosome segments will be common, and proportional to the number of rounds of polyploidization and stabilization since divergence of the lineages of present-day species. Superimposed on this will be micro-scale divergence of local gene content, caused by a variety of mechanisms, which may be more strongly proportional to time since divergence. A prediction is that other plant species would also show conserved frameworks of genes, in largely non-rearranged segments. More closely related species would show a higher proportion of conserved genes in related chromosomal segments, but the sizes of those segments may not correlate with time since divergence.

The complement of genes contained within all plants appears to be well conserved, with a large proportion of genes in even the most distantly related species, *A. thaliana* and rice, being matched *via* predicted amino acid sequences of their products [14,16]. Therefore, the prospects are good for grouping related genes in all plant species. But are the functions of genes conserved? Although the homologies of over half of the genes predicted in *A. thaliana* genomic DNA sequence indicated a broad function, the exact biological roles and phenotypic manifestations of very few of these genes are known. Some homology matches were noted with human genes involved in processes that do not exist in plants, e.g. at4g00020 is a homologue of Brca2, which is involved in breast cancer susceptibility. The significance of such matches will often lie more in structural constraints on particular binding or catalytic domains of proteins, rather than in biological role within the organism as a whole. Systematic functional analysis programmes have been initiated, which aim to take a variety of approaches to assigning functions to the genes of *A. thaliana* [17]. Such analyses are challenging in plants due to their genetic redundancy (even *A. thaliana* appears to be a degenerate tetraploid) and lack of an efficient homologous recombination system for site-directed mutagenesis. Also, the subtleties of gene expression patterns, as well as the amino acid sequences of the products they encode, are key in defining the contribution of genes to plant evolution [4]. However, the availability of complete genome sequence data for *A. thaliana* makes systematic and global functional analyses feasible. This is the next priority for the *A. thaliana* research community.

### References

1. Bevan M, Bancroft I, Bent E, *et al.* 1998. Analysis of 1.9 Mb of contiguous sequence from chromosome 4 of *Arabidopsis thaliana*. *Nature* **391**: 485–488.
2. Cavell A, Lydiate D, Parkin I, Dean C, Trick M. 1998. A 30 centimorgan segment of Arabidopsis thaliana chromosome 4 has six collinear homologues within the Brassica napus genome. *Genome* **41**: 62–69.
3. *C. elegans* Sequencing Consortium. 1998. Genome sequence of the nematode *C. elegans*: a platform for investigating biology. *Science* **282**: 2012–2018.
4. Doebley J, Lukens L. 1998. Transcriptional regulators and the evolution of plant form. *The Plant Cell* **10**: 1075–1082.
5. Kowalski ST, Lan T, Feldmann K, Paterson A. 1994. Comparative mapping of *Arabidopsis thaliana* and *Brassica oleracea* chromosomes reveals islands of conserved organization. *Genetics* **138**: 499–510.
6. Lagercrantz U, Putterill J, Coupland G, Lydiate D. 1996. Comparative mapping in *Arabidopsis* and *Brassica*, fine scale genome collinearity and congruence of genes controlling flowering time. *Plant J* **9**: 13–20.
7. Leitch IJ, Bennett MD. 1997. Polyploidy in angiosperms. *Trends Plant Sci* **2**: 470–476.

Copyright © 2000 John Wiley & Sons, Ltd. 

8. Lin X, Kaul S, Rounsley S, *et al.* 1999. Sequence and analysis of chromosome of the plant *Arabidopsis thaliana*. *Nature* **402**: 761–768.
9. Martin W, Gierl A, Saedler H. 1989. Molecular evidence for pre-Cretaceous angiosperm origins. *Nature* **39**: 46–48.
10. Mayer K, Schuller C, Wambutt R, *et al.* 1999. Sequence and analysis of chromosome of the plant *Arabidopsis thaliana*. *Nature* **402**: 769–777.
11. Moore G, Devos KM, Wang Z, Gale MD. 1995. Grasses, line up and form a circle. *Curr Biol* **5**: 737–739.
12. Muller J. 1981. Fossil pollen records of extant angiosperms. *Bot Rev* **47**: 1–142.
13. Paterson AH, Lan T-H, Reischmann KP, *et al.* 1996. Towards a unified genetic map of higher plants, transcending the monocot–dicot divergence. *Nature Genet* **14**: 380–382.
14. Somerville C, Somerville S. 1999. Plant functional genomics. *Science* **285**: 380–383.
15. Tanksley SD, Ganal MW, Prince JP, *et al.* 1992. High density molecular linkage maps of the tomato and potato genomes. *Genetics* **132**: 1141–1160.
16. van Dodeweerd A-M, Hall CR, Bent EG, Johnson SJ, Bevan MW, Bancroft I. 1999. Identification and analysis of homologous segments of the genomes of rice and *Arabidopsis thaliana*. *Genome* **42**: 887–892.
17. Walbot V. 1999. Genes, genomes, genomics. What can plant biologists expect from the 1998 National Science Foundation Plant Genome Research Program? *Plant Physiol* **119**: 1151–1155.
18. Wolfe KH, Gouy M, Yang Y-W, Sharp PM, Li W-H. 1989. Date of the monocot–dicot divergence estimated from the chloroplast DNA sequence data. *Proc Natl Acad Sci USA* **86**: 6201–6205.

*The*

**Genomics**

*Website at Wiley*

**www.wiley.co.uk/genomics**

The **Genomics** website at Wiley is a new and **DYNAMIC** resource for the genomics community, offering **FREE** special feature articles and new information **EACH MONTH**.

Find out more about *Comparative and Functional Genomics*, and enter the **Free Prize Draw** for the chance to win a year's free subscription.

Visit the **Library** for hot books in Genomics, Bioinformatics, Molecular Genetics and more.

Click on **Primary Research** for information on all our up-to-the minute journals, including: *Genesis*, *Bioessays*, *Gene Function and Disease*, and the *Journal of Gene Medicine*.

Let the **Genomics** website at Wiley be your **guide** to genomics-related web sites, manufacturers and suppliers, and a calendar of conferences.

WILEY

1516

Copyright © 2000 John Wiley & Sons, Ltd. 

